# Book Review

**Published:** 1917-01

**Authors:** 


### BOOK REVIEW

### CARE OF PATIENTS.

CARE OF PATIENTS undergoing Gynecologic and Abdominal Procedures, before, during, and after operation, by E. E. MONTGOMERY, M. D., Professor of Gynecology in Jefferson Medical College, Philadelphia. 12mo. of 149 pages with 6 illustrations. Philadelphia and London; W. B. Saunders Company. 1916. Cloth, $1.25 net.

The entrance of this little book into the literature of surgery will fill a long-felt need among those whose duty it has been to prepare for abdominal and gynecological operations, as well as prove of practical help to nurses and interns in getting patients ready for surgical proceedings and in caring for them, in an effectual way, after their operations have been performed.

The well-rounded and otherwise complete sitting of an operating room for a given operation may be set at naught by the oversight of a nurse or intern in leaving out some more or less essential instrument or in failing to have remembered to prepare the patient in suitable fashion for the surgical ordeal—oversights which prevent the surgeon's best work and pour out on the patient needless discomfort in the post-operative course, if not actual dangers, that might have been easily avoided. The author has given those seeking such help, in this excellent little volume, the detailed information needed to fortify against these common blunders.

In dealing with post-operative measures it is note-worthy that, while adhering closely to recognized practices as applied to the common conditions arising from abdominal and plastic operations, the author has broken away from the many needless, so-called essentials in the post-operative nursing of patients and has preached a new gospel—suggestions that seem so plausible and carry such conviction that one immediately resolves to adopt them—thereby converting this uncomfortable stage of a patient's hospital stay into a markedly more satisfactory one·

The sequential arrangement of the subjects treated, the terse nature of its chapters and the splendid illustrations in which it abounds, make an attractive, workable book which can be highly commended to nurses and interns. Aside from the purely technical part of the volume there will be found interesting chapters dealing with all the common abdominal and gynecologic operations, written in such simplicity as to prove of more than ordinary value to the young surgeon in his early steps alone in the field of pelvic and abdominal surgery.

